# Evaluating the dependency of neutron spectra and absorbed dose rates on the collimation field size in fast neutron therapy

**DOI:** 10.1016/j.heliyon.2021.e08274

**Published:** 2021-10-29

**Authors:** A.M. Shehada, V.P. Krivobokov, V.M. Golovkov

**Affiliations:** School of Nuclear Science & Engineering, National Research Tomsk Polytechnic University, Tomsk, 634050, Russia

**Keywords:** Fast neutrons, Collimation system, Neutron therapy, Absorbed dose rate, MCNP simulations

## Abstract

The aim of this research was to investigate the relationship between the collimator aperture and fast-neutron flux, neutron-energy spectrum and absorbed dose rate. For remote therapy, rather large fluxes of fast neutrons are needed which can create dose levels in the tissues of at least 0.1 Gy/min with a source-patient distance of 1 m. Advantageously for these purposes, the ^9^Be(d, n) reaction was investigated with deuteron energy of 13.6 MeV. The mean energy of the outgoing neutrons was obtained using the code PACE 4 (LISE++) which gave the value of about 5.2 MeV. The maximum neutron flux was at an energy of about 2.5 MeV. Samples activation analysis was deployed to measure the neutron flux in the energy-region [0–14 MeV]. The experimental works were carried out using Al, Fe, Cu and Cd foils which installed on the collimator apertures. To investigate the neutron spectrum, fluxes, and dose rates absorbed at the position of patients, experiments were conducted for four different neutron irradiation-field sizes, which can be modified by the removable-polyethylene parts. Simulation results obtained by the code MCNP-4C and PACE4 (LISE++) were comparable with the experimental data to some extent with consideration of some uncertainties of PACE4 results. It can be concluded that the neutron flux is depended on the irradiation-field size where the neutron flux output for bigger aperture size was about +25% comparing with the smaller ones. These results could play a significant role in improving the neutron flux and optimizing the collimation system utilized in fast neutron therapy. In addition, this can lead to optimization of irradiation canals installed in the nuclear reactors which employed for production of medical isotopes, material testing and many other applications.

## 1 Introduction

The reason of supporting the utilization of neutrons for treatment is their radiobiological effectiveness (RBE). For the neutron energies provided by the cyclotron beam, 1/3 fewer doses are required to accomplish an indistinguishable clinical impact with neutrons compared with customary photons. Certain tumors are radio-resistant. They react ineffectively to ordinary photon treatment. In these cases, neutrons are more effective by the factor of three in RBE [1].

Sources of fast neutrons deployed for radiotherapy should meet both neutron energy and source quality requirements [2]. The basic energy requirement is based on the requirement for neutrons to enter the depth at which the tumor is located without a harmful amount of the dose of neutrons absorbed into the healthy tissue through which they pass. If the neutron energy is too low, the dose of radiation on healthy tissue is so high that healthy tissue may not recover. Radiation therapists, who have extensive experience using beams from ^60^Co sources and good knowledge about their tissue attenuation, may wish to have a neutron source that gives equivalent or better tissue penetration.

With a specific end goal to restrict the treatment volume, a collimator must be utilized. In the event that the source is too substantial, the length of the collimator is troublesome and an unwanted penumbra is presented. The collimator can enhance the neutron flux at a given separation from the source where the collimator scatters neutrons into the collimated beam. How much increment happens relies upon the collimation-field and the length of the collimator. On the other hand, when the neutron source is located at far distance, the collimator could lessen the neutron flux as opposed to increase it. In most cases, the collimator can improve the neutron flux up to 20%.

Many works dedicated to measuring the yield, energy, and dose rate of neutrons emitted by the reaction ^9^Be(d,n) [3, 4, 5]. The neutron yield for our deuteron energy 13.6 MeV is about 2.5 × 10^10^ neutrons sr^−1^μC^−1^. The average neutron energy for the same deuteron energy 13.6 MeV is about 5 MeV.

For fast neutrons, the average absorbed dose rate given in gray per second, from neutrons with mean energies *E*_*n*_, can be obtained using the following equation [6]:

(1) $$\text{D}{\;}_{\left(\text{Gy/s}\right)}=\frac{\phi E_{n}\;\sum_{i}N_{i}\sigma_{i}f_{i}}{1\frac{J}{Kg}/Gy}$$

where *φ* (n/cm^2^/s) is the neutron flux of energy *E*_*n*_. While, *N*_*i*_ is the number of atoms per kilogram of the *i-*element, *σ*_*i*_ is the scattering cross-section of the *i-*element for neutrons of energy E, in cm^2^, and *f*_*i*_ is the maximum fractional energy transferred from neutrons to the scattered atom of *i-*element during the collision.

The maximum energy transferred *f*_*i*_ is given by the equation [7, 8]:

(2) $${\text{f}}_{\text{i}}=\frac{4M_{i}m_{n}}{{\left(M_{i}+m_{n}\right)}^{2}}$$

where *M*_*i*_ and *m*_*n*_ are the masses of the nucleus and neutron, respectively. By using the values from [9, 10, 11, 12], one can calculate the absorbed dose rate for the human tissues.

## 2 Experiment and method

### 2.1 Collimator setup

**Figure 1:**
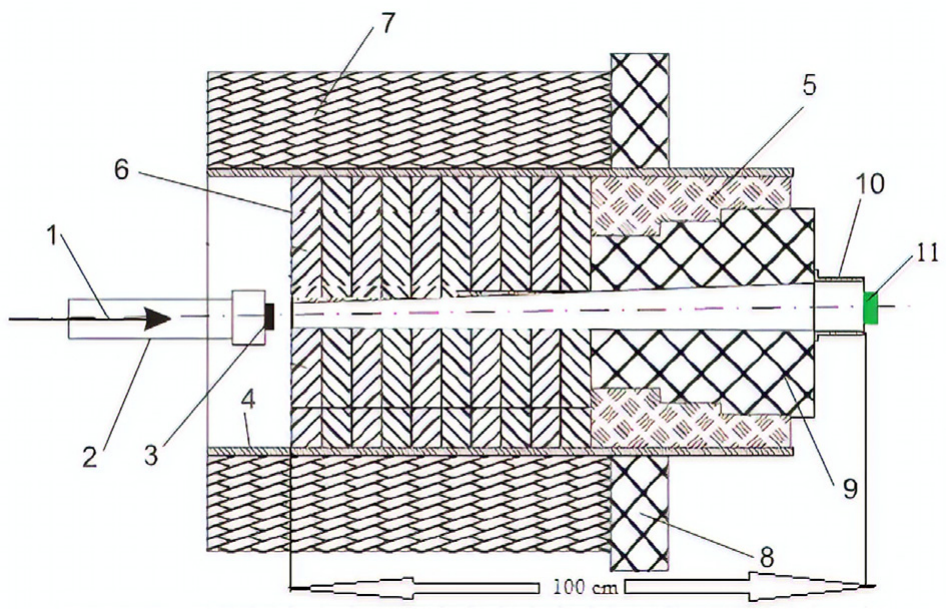
The detailed structure of the collimator; 1 – deuteron beam; 2 – ion beam channel; 3 – Be-target; 4 – iron pipe; 5 – polyethylene collimator; 6 – iron disks; 7 – concrete wall; 8 – radiation protection of polyethylene; 9 – removable polyethylene collimator; 10 – cone; 11 – detection foils.

Four different collimator irradiation fields have been used with removable-polyethylene collimators which have different irradiation-field sizes; 8.5 × 8.5 cm^2^, 10.5 × 6 cm^2^, 7 × 4.5 cm^2^ and 4.5 × 4.5 cm^2^. The geometry of collimator materials and shielding are shown in Figure 1. Neutron activation method was deployed using Al, Fe, Cu, and Cd foils to measure the real values of neutron flux and study the influence of collimator aperture size on neutron flux and spectrum. Aluminum foils were used with dimensions 3.7 cm × 0.75 cm, thickness 0.2 cm, and a mass of 2 g, while the iron foils have cylindrical shape with 2 cm diameter, 1 cm in thickness, and a mass of 25 g. The Cu and Cd foils were 2 × 2 cm^2^ in dimensions and 0.1 mm in thickness. The length of the collimation system from the beginning of the iron blocks (which is separated 5 cm from the Be target) to the end of the plastic cone is 100 cm, as shown in Figure 1. The detection samples were fixed at the aperture of the collimator and centered on the collimator axis. The collimator canal is open to air as the aperture is not sealed and always changing the aperture field size.

### 2.2 Neutron detection

**Figure 2:**
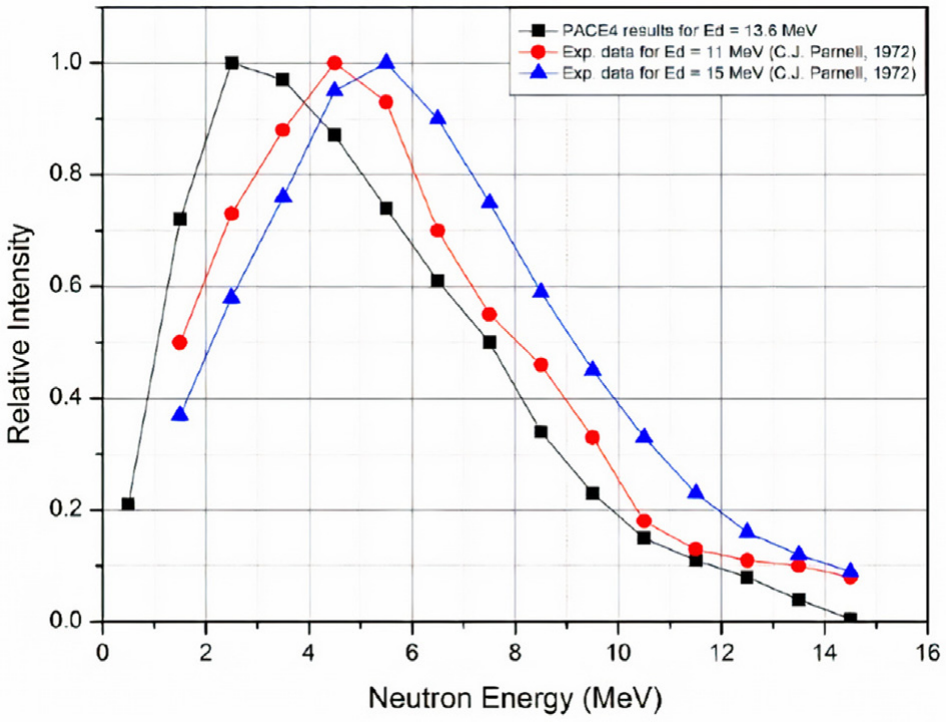
The simulation results of the code PACE 4 for the spectrum of neutrons emitted by the reaction Be^9^(d, n) for E_d_ = 13.6 MeV and emission angle 0^o^ along with the data from reference [5].

**Figure 3:**
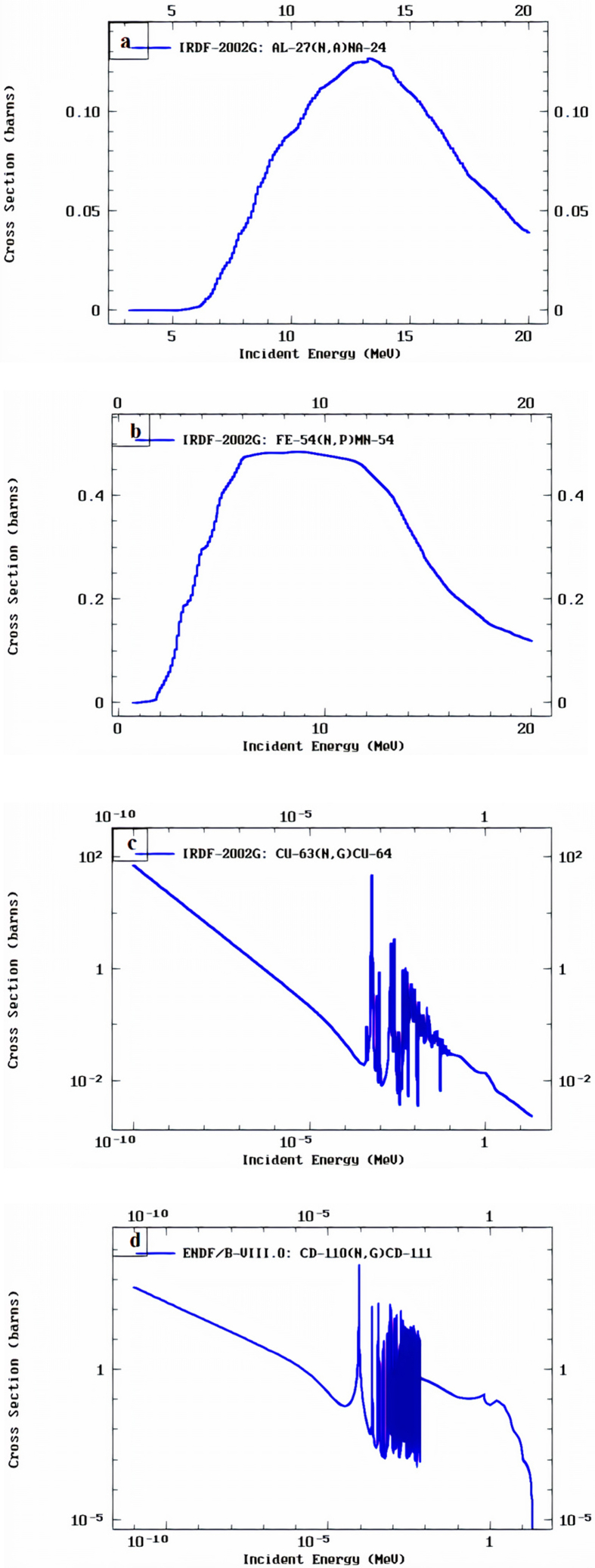
The neutron interaction cross-sections of a: ^27^Al (n, a), b: ^54^Fe (n, p), c: ^63^Cu (n, γ), and d: ^110^Cd (n, γ), from IRDF-2002G library [14, 15] and ENDF/B-VIII.0 library [16] respectively.

Beryllium target with 5 cm in diameter and 2 mm thickness has been irradiated by deuteron ions with energy 13.6 MeV for a period of 30 min and current ∼45 μA where the spot size was about ∼15–20 mm. The energy spectrum of neutrons was simulated and obtained by the code PACE 4 (LISE++) [13] as shown in Figure 2. The results then were adopted in MCNP-4C simulations. The energy spectrum showed that the peak of neutron flux is at about 2.5 MeV of neutron energy, while the mean neutron energy is about 5.16 MeV. This energy spectrum encourages the use of Al-27 and Fe-54 foils as detectors for this range of neutron energy as shown from the interaction cross-sections curves in Figure 3. The radioactivity of formed isotopes ^24^Na (T_1/2_ = 14.96 h) and ^54^Mn (T_1/2_ = 312.3 d) through the reactions ^27^Al(n,α) (0.12 barn) and ^54^Fe(n, p) (0.6 barn) were measured. The emitted photons have an energy of 1369 keV (100 %) for ^24^Na and 834.85 keV (99.976 %) for ^54^Mn. For epithermal neutrons (0.4 eV–1 MeV), a copper foil was used on which the reaction ^63^Cu (n,γ) ^64^Cu (σ = 4.5 barn, T_1/2_ = 12.7 h) takes place. The isotope ^64^Cu ends up to ^64^Ni through ec β^+^ emission (61.5%), and gamma-ray photons produced by β^+^ with energy of 511 keV (34.8 %). The cadmium foils were used for the thermal-neutrons region, due to reaction ^110^Cd (n,γ) ^111^Cd (σ = 11 barn,T_1/2_ = 48.5 m), which emits gamma rays with energies 245.4 keV (94 %) and 150.8 keV (29.1 %). The areas under the photo-peaks were calculated for each foil, and after making all the standard corrections, the neutron spectra and fluxes for each collimator were calculated. The neutron flux emitted by the beryllium target was measured by Al and Fe foils and gave a value of 5.3 × 10^9^ neutrons cm^−2^s^−1^ (or 2.3 × 10^10^ neutrons sr^−1^μC^−1^) for deuteron-beam current equal to 45 μA, which agrees with [3].

The code PACE is a modified version of JULIAN – the Hillman–Eyal evaporation code using a Monte-Carlo code coupling angular momentum. From Figure 2 it can be concluded that there are some differences between PACE4 results and the experimental data, which are obvious for the maximum energy of neutrons. This inconsistency may be due to the restrictions of models in the PACE code especially for light elements. On the other hand, the two average energies are close in value and the neutron average energy is the important component used in Eq. (1) when calculating the absorbed dose rate. In addition, there are no significant differences in the values of conversion coefficients of fluence-to-effective dose for neutrons with energies between 2.5 and 5 MeV, which gives almost the same values for the effective dose in this energy-range [17]. Therefore, when dealing with energy spectrum, the mean energy is the considered value for absorbed dose calculations.

The radioactivity of isotopes was obtained by using a detector of high-purity coaxial germanium HPGe (GC1020). The counting time was 250 s for Al, Cu, and Cd foils and 4000 s for Fe foils. The efficiency of this detector for gamma-rays with energies of 834.85 keV and 1369 keV are about 0.6% and 0.35%, respectively. The measured neutron flux was calculated by using the following equation:

(3) $$\varphi \text{ ​}\left({\text{n.cm}}^{-2}{\text{.s}}^{-1}\right)=\frac{A}{<\sigma >N\left(1-e^{-\lambda t_{irr}}\right)e^{-\lambda t_{c}}}$$

where *A, λ, <σ>, N, t*_*irr*_*,* and *t*_*c*_ are the under-peak area activity (Bq), decay constant (s^−1^), effective cross section (cm^2^), number of target nuclei, irradiation time, cooling time, respectively.

## 3 Results and discussion

**Figure 4:**
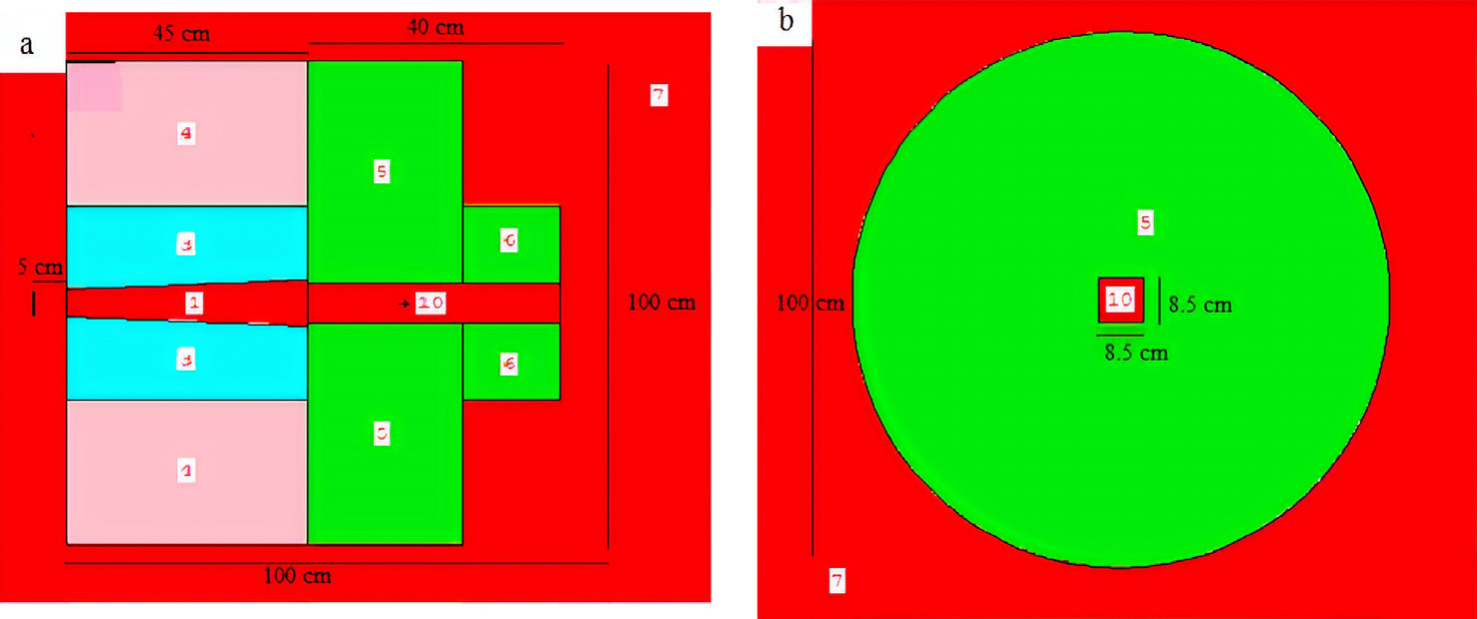
The MCNP geometry of collimator with 8.5 × 8.5 cm^2^ aperture. The numbers inside the images refer to cells (parts of the collimator and their materials). a; represents the view according to X–Y plan, b; represents the view according to Y-Z plan.

**Figure 5:**
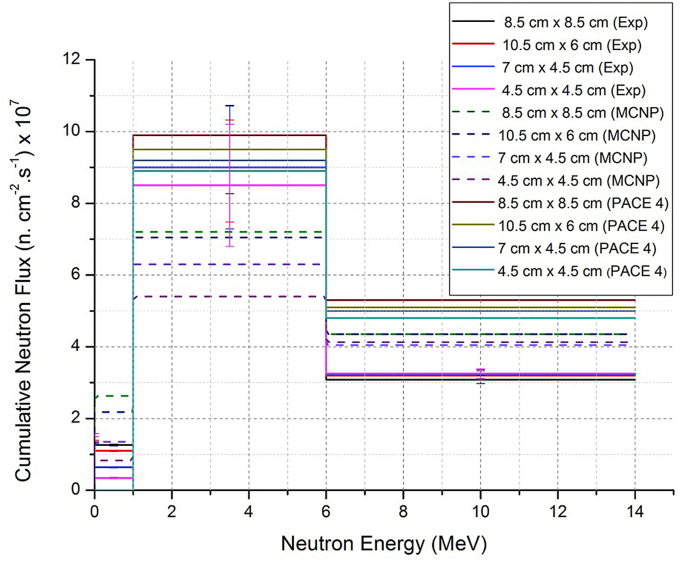
Cumulative neutron fluxes over energy regions of neutrons for different collimation irradiation fields. This scheme is presenting data obtained experimentally (with error bars) and simulation results by MCNP-4C and PACE 4 codes.

**Figure 6:**
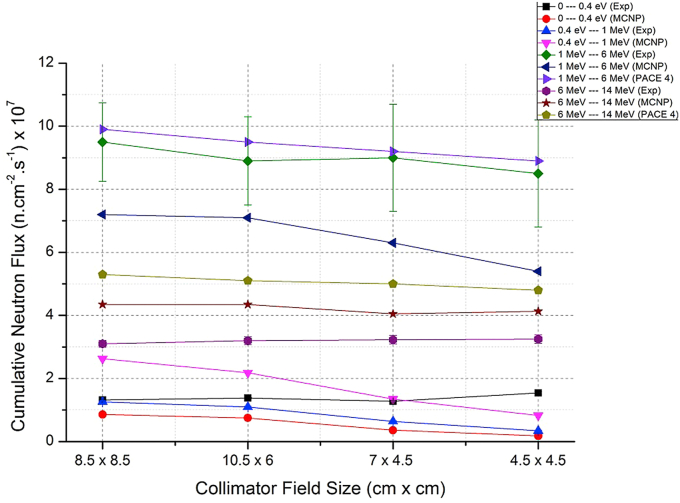
Cumulative neutron fluxes for different collimation irradiation fields and neutron energy regions, presenting experimental, MCNP-4C and PACE 4 codes results.

The extracted results from experiments and MCNP-4C simulations for neutron fluxes in different energy ranges of neutrons and the neutron doses absorbed are presented in Tables 1 and 2. These results beside the results obtained by PACE 4 code were compared in Figures 5 and 6. Figure 4 demonstrates the MCNP geometry of the collimator.

**Table 1:** The experimental and MCNP-4C results of neutron fluxes measured by neutron activation method of the foils Fe, Al, Cu, and Cd for different collimator irradiation fields.

Sample	Collimator (cm x cm)	Experimental Neutron Flux (Error %) (n/cm^2^ s) x 10^7^	Neutron flux by MCNP4C Code (Error <5 %) (n/cm^2^ s) x 10^7^
For iron foils which respond to neutron energy range 1 MeV–14 MeV (Fast Neutrons)			
Fe 1	8.5 × 8.5	12.54 (13 %)	11.55
Fe 2	10.5 × 6	12.09 (16 %)	11.40
Fe 3	7 × 4.5	12.27 (19 %)	10.35
Fe 4	4.5 × 4.5	11.72 (20 %)	9.53
For Aluminum foils which respond to neutron energy range 6 MeV–14 MeV (Fast Neutrons)			
Al 1	8.5 × 8.5	3.08 (3.5 %)	4.35
Al 2	10.5 × 6	3.20 (3.8 %)	4.35
Al 3	7 × 4.5	3.23 (3.9 %)	4.05
Al 4	4.5 × 4.5	3.25 (4.1 %)	4.13
For Copper foils which respond to neutron energy range 0.4 eV–1 MeV (Epithermal Neutrons)			
Cu 1	8.5 × 8.5	1.26 (2.2 %)	2.63
Cu 2	10.5 × 6	1.10 (2 %)	2.18
Cu 3	7 × 4.5	0.64 (2.7 %)	1.35
Cu 4	4.5 × 4.5	0.34 (5.6 %)	0.83
For Cadmium foils which respond to neutron energy range 0 eV–0.4 eV (Thermal Neutrons)			
Cd 1	8.5 × 8.5	1.32 (1.4 %)	0.86
Cd 2	10.5 × 6	1.38 (1.4 %)	0.75
Cd 3	7 × 4.5	1.28 (1.9 %)	0.36
Cd 4	4.5 × 4.5	1.54 (2.6 %)	0.18

**Table 2:** Experimental and MCNP-4C results of neutron absorbed dose rates at 1 m from the source for different collimator irradiation fields.

	Neutron absorbed dose rates in Gy/min at 1 m from the source			
	8.5 × 8.5 cm^2^	10.5 × 6 cm^2^	7 × 4.5 cm^2^	4.5 × 4.5 cm^2^
Exp. results	0.225	0.216	0.21	0.20
MCNP-4C_results	0.225	0.217	0.191	0.17

From Table 1 and Figure 3, the Fe foils were suitable for fast-neutron detection in the neutron energy-range 1 MeV–14 MeV, and Al foils were suitable for fast-neutron detection in the energy-range between 6 MeV to 14 MeV. Whereas, Cu and Cd foils were good detectors for epithermal and thermal neutron energy regions, respectively. As a result, it is noticeable that the neutron flux in the energy range [1 MeV–6 MeV] is the most contributed component of the total neutron flux and represents about 1.5–2 times the neutron flux in the energy range [6 MeV–14 MeV]. These results are expected as previously predicted and calculated by PACE 4 and MCNP-4C codes where the average neutron energy is 5.2 MeV and the maximum at energy about 2.5 MeV as shown in Figures 2, 5, and 6.

The absorbed dose rates vary depending on the collimation field size. As shown in Table 2, the experimental and MCNP-4C values were almost equal for large collimation apertures, and begin to disagree for smaller ones. The absorbed dose rate was decreased by about 10% when comparing between the collimation apertures 8.5 × 8.5 cm^2^ and 4.5 × 4.5 cm^2^ as concluded from the experimental results, and decreased by 25 % as calculated by the MCNP-4C code. This result can be explained as appeared in Figures 5 and 6, where the neutron flux calculated by MCNP-4C is about 10% less than neutron flux measured experimentally in the energy-range 1 MeV–6 MeV, taking into account the large uncertainties of 13–20 % for some experimental results. The absorbed dose rate of neutrons emitted by reaction ^9^Be (d, n) at 1.25 m from source can be calculated using the equation: $\overset{•}{D}=\text{ ​}0.000124\text{ ​}{\text{E}}_{d}^{2.99}$[4, 5], where E_d_ is the deuteron energy in MeV and $\overset{•}{D}$ is in rad μA^−1^min^−1^. For deuteron energy E_d_ = 13.6 MeV and current 45 μA, this equation gives a value of 0.215 Gy/min at 1 m from a source, which is comparable to our result 0.225 Gy/min at 1 m in the case of collimation field size 8.5 × 8.5 cm^2^.This equation was obtained from fitting the data of [4] for the dose rate of fast neutrons in tissue as a function of deuteron energy on thick beryllium target. The aperture size was 5 × 5 cm at a distance of 1.25 m. A power law formula was chosen to compare with the results of [5]. For neutrons with energies more than 20 MeV, an obvious disagreement between the results of this equation and those reported by [5], and this discrepancy increases with increasing energy.

The discrepancies between experimental results and the MCNP simulation results are mainly due to the uncertainties in the reaction cross-section of Cu-63(n,g) in the neutron energy range [0.4 eV to 1 MeV]. The assumed value of reaction (n,g) cross section was 4.5 barns as found from the library IRDF-2002G, but this value is related to thermal neutrons and not extending to the energy range till 1 MeV for the Cu-63(n,g) reaction. So, this value has a large uncertainty and thus large discrepancies appeared in this energy region. From this result and from Eq. (3), it was obvious that the effective cross-section for Cu-63(n,g) for the neutron energy range [0.4 eV to 1 MeV] should be about half the value used in the calculations (about 2.25 barns) to match the results of MCNP simulations. On the other hand, the discrepancies between experimental results and simulation results in the thermal region represented by the Cd-110(n,g) reaction, could be also related mainly to the cross-section uncertainty.

The difference between the experimental and simulated results in the neutron energy range 1–6 MeV and between 6 to 14 MeV is mainly due to the neutron spectrum that was adopted in this study and which is based on the results of the simulation of the PACE4 program as shown in Figure 2. Whereas, compared to the results of previous experiments [5], it is clear that the spectrum in the case of PACE4 was shifted towards lower energies by about 2.5 MeV. By taking into account these corrections, an agreement can be obtained between the experimental values and the MCNP simulations, taking into account also the experimental uncertainties in the energy-region 1–6 MeV, which are about 13–20%. But in general, this will not affect the calculations of the radiation dose rate significantly because the value entered in Eq. (2) is the average energy of neutrons. The average energy of neutrons calculated using the code PACE4 is about 5.2 MeV, and the value resulting from some previous experiments is about 5 MeV, as in reference [3].

## 4 Conclusions

Both experimental and simulation works deploying MCNP4C and PACE4 codes were carried out to investigate the dependency of neutron-energy spectrum, neutron flux and the absorbed dose rate on the collimation apertures of collimators constructed for fast neutron therapy. The obtained results were in good agreement with other related literatures which concluded that the neutron flux can be dependent on the collimation and irradiation aperture size and shape. From this study, the experimental and simulation results showed that the neutron flux and absorbed dose rate are dependent on the collimation aperture size. The simulation and experimental results were in good agreements for big collimation apertures, and slightly began to disagree for the smaller ones. It was found experimentally that there is about 10% difference of absorbed dose rate between the collimation apertures 8.5 × 8.5 cm^2^ and 4.5 × 4.5 cm^2^, while for the MCNP4C simulation results the difference was approximately 25%. The discrepancy between experimental and MCNP results were mainly related to the underestimated neutron energy spectrum simulated by the code PACE4 and adopted in MCNP simulations. Therefore, MCNP results showed bigger difference than experimental results when comparing between results of the collimators 8.5 × 8.5 cm^2^ and 4.5 × 4.5 cm^2^. So, it is recommended to carefully not rely entirely on the results of PACE4 code when calculating fast neutron spectrum for some reactions or using them with limitations. As a conclusion, this work may lead to better way to optimize the collimation and irradiation-field and maximize the fast-neutron flux delivered to the patient and minimizing the time of treatment when using the appropriate collimator size.

## Declarations

### Author contribution statement

Abdullah Mohammad Shehada: Conceived and designed the experiments; Performed the experiments; Analyzed and interpreted the data; Contributed reagents, materials, analysis tools or data; Wrote the paper.

Krivobokov Valery Pavlovich: Conceived and designed the experiments; Performed the experiments.

Golovkov Vladimir Mikhalovich: Conceived and designed the experiments; Performed the experiments; Analyzed and interpreted the data; Contributed reagents, materials, analysis tools or data.

### Funding statement

This work was supported by the National Research Tomsk Polytechnic University under contract No. 23321; 05.12.2018.

### Data availability statement

The data that has been used is confidential.

### Declaration of interests statement

The authors declare no conflict of interest.

### Additional information

No additional information is available for this paper.
